# The role of MMP-14 in ovarian cancer: a systematic review

**DOI:** 10.1186/s13048-021-00852-7

**Published:** 2021-08-03

**Authors:** M. Caroline Vos, Anneke A. M. van der Wurff, Toin H. van Kuppevelt, Leon F. A. G. Massuger

**Affiliations:** 1 Department of Obstetrics and Gynaecology, Elisabeth-Tweesteden Hospital, PO Box 90151, 5000 LC Tilburg, the Netherlands; 2 Department of Pathology, Elisabeth-Tweesteden Hospital, PO Box 90151, 5000 LC Tilburg, the Netherlands; 3 Department of Biochemistry, Radboud Institute for Molecular Life Sciences, Radboud University Medical Centre, PO Box 9101, 6500 HB Nijmegen, the Netherlands; 4 Department of Obstetrics and Gynaecology, Radboud University Nijmegen Medical Centre, PO Box 9101, 6500 HB Nijmegen, the Netherlands

**Keywords:** MMP-14, Ovarian cancer, Review, Cancer pathophysiology, Immunohistochemistry

## Abstract

**Aim:**

In order to evaluate the role of MMP-14 in ovarian cancer, a systematic review was conducted.

**Methods:**

In March 2020, a search in Pubmed was performed with MMP-14 and ovarian cancer as search terms. After exclusion of the references not on MMP-14 or ovarian cancer or not in English, the studies found were classified into two categories: basic research and clinicopathological research.

**Results:**

In total, 94 references were found of which 33 were excluded. Two additional articles were found in the reference lists of the included studies. Based on the full texts, another 4 were excluded. Eventually, 59 studies were included in the review, 32 on basic research and 19 on clinicopathological research. 8 studies fell in both categories. The basic research studies show that MMP-14 plays an important role in ovarian cancer in the processes of proliferation, invasion, angiogenesis and metastasis. In clinocopathological research, MMP-14 expression is found in most tumours with characteristics of poor prognosis but this immunohistochemical MMP-14 determination does not seem to be an independent predictor of prognosis.

**Conclusions:**

From this systematic review of the literature concerning MMP-14 in ovarian cancer it becomes clear that MMP-14 plays various important roles in the pathophysiology of ovarian cancer. The exact translation of these roles in the pathophysiology to the importance of MMP-14 in clinicopathological research in ovarian cancer and possible therapeutic role of anti-MMP-14 agents needs further elucidation.

**Supplementary Information:**

The online version contains supplementary material available at 10.1186/s13048-021-00852-7.

## Introduction

Ovarian cancer is known for its poor prognosis, due to the lack of effective screening methods and, therefore, its detection is usually in an advanced stage. Despite intensive treatment with surgery and chemotherapy and emerging options using targeted agents, ovarian cancer is still the leading cause of gynaecological cancer-related death in Europe and the United States. (http://gco.iarc.fr/today/home) [1]

In search for new targets for treatment, matrixmetalloproteinases (MMPs) seem an attractive option. Members of the matrix metalloproteinase (MMP) family, also known as matrixins, belong to the metzincin superfamily. They are involved in the breakdown of extracellular matrix, not only in normal physiological processes, but also in pathological processes such as inflammation and cancer. MMPs are characterized by their zinc-binding site and the necessity of the containment of zinc for their enzymatic action [2].

Apart from their action as collagenases, gelatinases and stromelysins, various other roles for MMPs in cancer have been discerned. They have an effect at the tumour-cell level, intracellular actions in the nucleus [3] and in epithelial-to-mesenchymal transition and proliferation [4] and at the tumour micro-environment level, involvement in invasion, angiogenesis and metastasis [5]. In the context of inflammation, they influence T-cell inhibition and adhesion and macrophage inhibition [6]. Known and registered inhibitors of MMPs include the tetracyclines, of which doxycycline has been well studied [7]. Based on these results, the role of MMPs in ovarian cancer should be investigated further [8, 9]. So far however, no therapeutic effect has been demonstrated for MMP inhibition in ovarian cancer [10, 11]. Since then, several new targeted agents against MMPs have been developed including antibodies and one of these new agents is a MMP-14 specific nanoprobe that facilitates in vivo detection of MMP-14 tumour cells. After administration of the nanoprobe, the tumour cells with nodules as small as 125 μm can be made visible with fluorescence [66].

In this review, we focus on MMP-14 (formerly identified as MT1-MMP) [12]. Most MMPs are secreted as inactive proproteins, which are activated when cleaved by extracellular proteinases [2]. However, MMP-14 is a member of the membrane-type MMP (MT-MMP) subfamily, which is characterised by a transmembrane domain, so that the MMPs are expressed at the cell surface rather than secreted [2]. In 1994, MMP-14 was the first membrane-bound MMP to be described [12] and its role on invasion and metastasis has been demonstrated in animal models [13, 14] Also, MMP-14 predicts prognosis in cancer in general according to a recent review [15].

MMP-14’s main substrates are pro-MMP-2 and collagen I, but collagen II and III can also be cleaved be it to a lesser extent [2]. MMP-14 forms a dimer at the cell surface and a complex with MMP-2 and TIMP-2 (Tissue Inhibitor of MetalloProteinases 2) in order to activate MMP-2 [16–18]. It is also an enzyme for degradation of gelatine and cleavage of CD44 (a hyaluronan-receptor) [19].

MMP-14 expression varies depending on cancer type and is high in mesenchymal tumours, melanomas and brain tumours [4] and also found in hepatic tumours and in carcinomas including breast cancer [20, 21]. Though MMP’s and ovarian cancer were reviewed before, [22] no systematic review or meta-analysis on the role of MMP-14 alone in ovarian cancer has yet been published.

## Methods

On 23 March 2020, we conducted a search in Pubmed on ‘MMP-14 AND ovarian cancer’. For details of the search strategy, see Additional file 1: Appendix 1.

The following exclusion criteria were used: the study was not MMP-14 or ovarian cancer or the study was not in English. After screening titles and abstracts for relevance and excluding studies on other topics, full texts of the remaining studies were assessed according to the same inclusion and exclusion criteria. By screening the reference lists of those full texts, additional references were detected and added to the search.

## Results

By searching on ‘MMP-14 AND ovarian cancer’, a total of 94 references were found, of which 33 were excluded after screening of the titles and abstracts. The following exclusion criteria were determined: 18 studies were not on MMP-14, 11 studies were not on ovarian cancer and 4 studies were in Chinese.

Screening the reference lists of the remaining 61 studies yielded two additional articles on ovarian cancer and MMP-14.

**Fig. 1:**
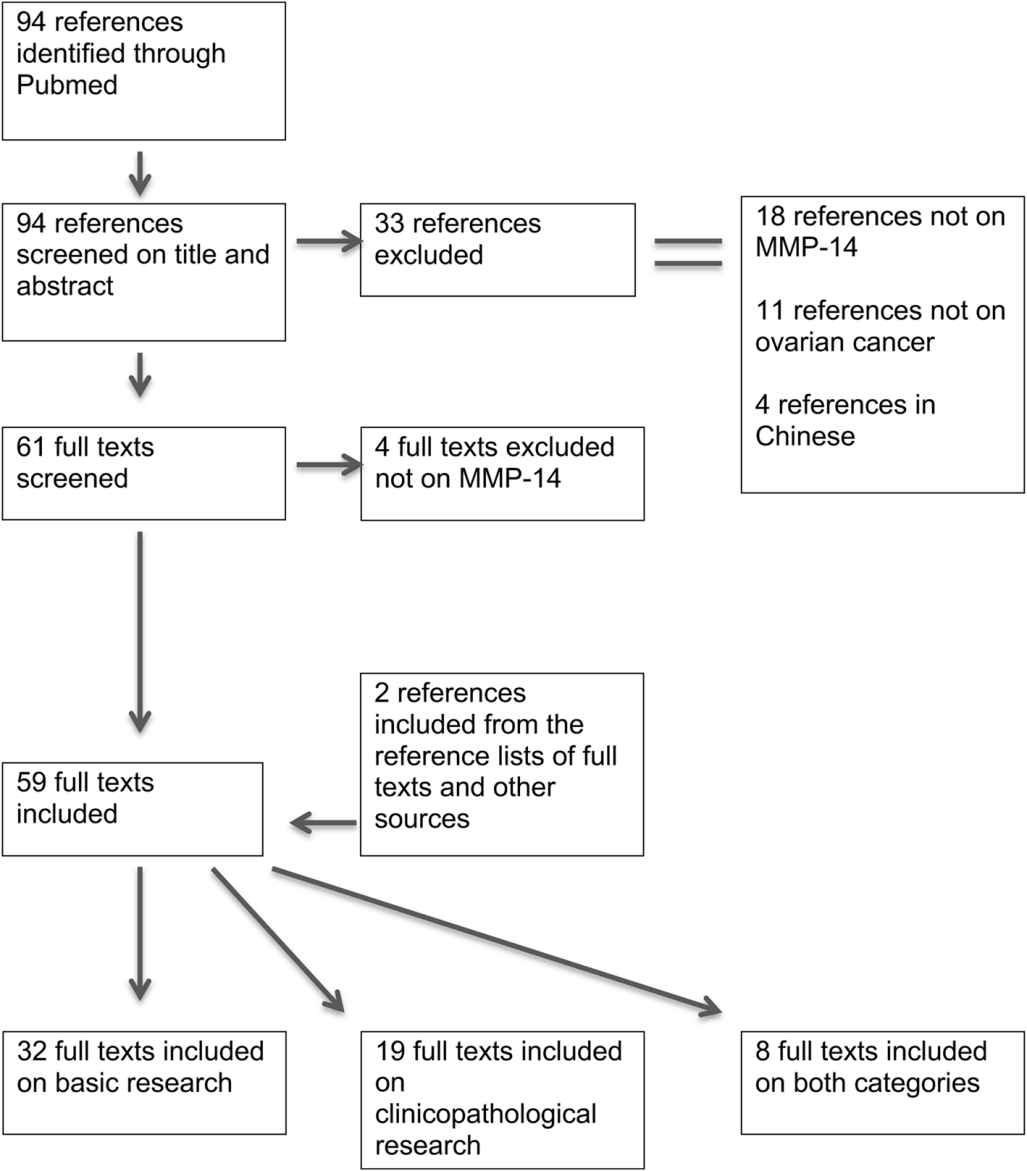
Flowchart of the study

After examining the full texts of the 63 articles, another four were excluded because the subject was only remotely on MMP-14. Eventually, 59 articles were included in this review on MMP-14 and ovarian cancer: 32 on basic research, 19 on clinicopathological research and 8 in both categories. For a flowchart of the results of the search strategy, see Fig. 1.

### MMP-14 and ovarian cancer in basic research

The first study on the role of MMP-14 in ovarian cancer was performed in 1996 [23]. With Western blotting, immunohistochemistry (IHC) and immunoprecipitation, Fishman et al. demonstrated the presence of MMP-14 on the surface of cultured ovarian cancer cells derived from primary ovarian tumours, metastatic tissue and ascites.

#### Development of cell lines, antibodies and MMP-inhibitors

A number of studies primarily reported on the development of materials that were used: cell lines, antibodies and MMP-inhibitors [24–28]. These studies also reported that more MMP-14 expression in the developed cell lines results in more invasion in vitro.

#### Proliferation

There is scant evidence of the effect of MMP-14 expression on proliferation in ovarian cancer. In a model with human ovarian tumours in SCID mice, Drew et al. found that MMP-14 mRNA expression from both human tumour and mouse stromal cells was associated with increased tumour size, whereas only MMP-14 mRNA expression by human tumour cells was associated with tumour spread [29]. By cloning an antisense MMP-14 into SW626 ovarian cancer cells, Wu et al. induced decreased proliferation of this cell line [30]. Liao et al. investigated hedgehog (HH) pathway proteins, finding that increased Gli1 expression is correlated with increased MMP-14 expression and increased proliferation and invasiveness. By using inhibitors of HH pathway proteins, this effect was reversed and MMP-14 expression reduced [31]. Moss et al. demonstrated increased proliferation of tumour cells in three-dimensional collagens gels as a result of MMP-14 expression [32]. Koshikawa demonstrated that MMP-14 promotes heparin-binding EGF-like growth-factor-induced proliferation of ovarian cancer cells both in suspension and in collagen gel [33]. As demonstrated in a 3D collagen model, hypoxia induces MMP-14 dependent proliferation [34]. From the various models in this limited number of studies, it can be concluded that MMP-14 seems to play a role in an increase in proliferation of ovarian cancer cells.

#### Invasion

The first study [23] was followed by a series of studies on invasion. In the above-mentioned mouse model, Drew et al. showed that MMP-14 mRNA and protein expression by the tumour cells was associated with tumour spread intra-peritoneally [29].

MMP-14 was shown to induce activation of MMP-2 in DOV13 cells cultured on three-dimensional collagen lattice stimulated by β_1_ integrins [35]. Further interactions between type-I collagen, α_2_β_1_ and α_3_β_1_ integrins, MMP-14, MMP-2 and TIMP-2 were demonstrated [36], as well as promotion by lysophosphatidic acid (LPA) [37]. Early growth response protein-1 (EGR1), but not EGR-2–4, activates MMP-14 [38]. Lee et al. demonstrated that degradation of collagen by invasive cell lines is mediated by MMP-14 through phagocytosis [39]. This mechanism is different from the mechanism described earlier [35, 36], though the apparent differences may be attributable to the models that were used. Agarwal et al. demonstrated that MMP-14 and MMP-9 activate pro-MMP-1-dependent migration of OVCAR4 cells [40]. Hypoxia not only induces proliferation, but also MMP-14-dependent invasion [34]. Anti-sense MMP-14 cloned into SKOV3 cells showed a lower percentage of invasion, as well as inhibited activation of pro-MMP2 [41]. The same antisense vector was used in SW626 cells, again demonstrating the importance of MMP-14 for proliferation and invasion [30].

In contrast to these positive results on the importance of MMP-14 in invasion, Klymenko et al., in a study on cadherins, showed that mesenchymal N-cadherin-positive cells and MCAs (MultiCellular Aggregates) invade more effectively than E-cadherin-expressing cells [42]. In this study, the application of a (broad) MMP-inhibitor did not inhibit invasion fully. In a study on the application of cisplatin and a mammalian target of rapamycin (mTOR) inhibitor, invasion and migration of tumour cells was drastically impaired [43]. The expression of MMP-14 remained unchanged, but the level of activated MMP-2 was drastically reduced.

In a study on invasion and metastasis, an anti-MMP-14 antibody inhibited invasion and metastasis both in vitro and in vivo. In the in vivo mouse model intraperitoneal administration of anti-MMP-14 was combined with docetaxel, and this treatment reduced invasion of the diaphragm and development of thoracic metastases [44].

Combined, most in vitro and in vivo studies demonstrated an increase of invasion in the presence of MMP-14.

#### Angiogenesis

Only a limited number of studies report on the role of MMP-14 in angiogenesis in ovarian cancer. In a study on vasculogenic mimicry, Sood et al. demonstrated the formation of matrix-rich tubular networks by culturing ovarian cancer cell lines in Matrigel [45] By treating the samples with an MMP-inhibitor, the formation of the tubular networks was suppressed. Leroy-Dudal et al. demonstrated the ability of IGROV1 and SKOV3 cells to transmigrate through HUVEC (human umbilical vein endothelial cells)-derived ECM (endothelium-derived extracellular matrix) in vitro, [46] where αν integrins, MMP-14 and MMP-2 play an important role.

In a nude mouse model with OVCAR-4 or SKOV-3 cells, treatment with cell-penetrating pepducins targeting this cascade resulted in inhibition of PAR1-dependent angiogenesis, ascites and metastasis [40]. In another mouse model, MMP-14 was found to induce angiogenesis, and this effect was inhibited by an anti-MMP-14 antibody [44]. These few studies thus report a positive effect of MMP-14 on angiogenesis and a negative effect of MMP-14-inhibition.

#### Metastasis

Many of the mechanisms of invasion described above are also relevant to metastasis.

MMP-14 plays a key role in detaching cells from the primary tumour and forming multicellular aggregates (MCA) that subsequently adhere to and invade mesothelial monolayers [32]. These MCAs exhibit more aggressive behaviour than single cells. The submesothelial matrix is rich in collagen type I and III, and MMP-14 is necessary for adherence to and invasion in this matrix. The post-translational phosphorylation of the cytoplasmatic tail of MMP-14 at Thr^567^ influences the behaviour of individual cells and MCAs. Enhanced phosphorylation results in detachment of cell–cell adherent sheets and increased cell migration [32]. The MCAs with enhanced phosphorylation show increased adherence to peritoneum and this adherence degrades mesothelial integrity [47]. Co-expression of integrin-linked kinase (ILK) and MMP-14 results in phosphorylation of the cytoplasmatic tail of MMP-14 with increased cell–cell detachment, migration, adherence to the peritoneum and degradation of mesothelial integrity [48].

#### Interactions of MMP-14 with other molecules

Several studies have investigated the interactions between MMP-14 and other molecules. From the very first study onwards, the action of MMP-14 as the main activator of MMP-2 has repeatedly been demonstrated by zymography, as have the presence and inhibitory effect of TIMP-2 [23, 35].

A very interesting finding is the inverse relationship between MMP-14 and MUC16/CA-125. CA 125 is an antigen which is known for its diagnostic and prognostic value in ovarian cancer. MMP-14 overexpression causes increased ectodomain shedding of MUC16/CA 125. By the increased ectodomain shedding of MUC16/Ca 125, integrins on the cell surface may become exposed resulting in increased adhesion and invasion [49]. The interactions between MMP-14 and the integrins have been extensively studied as discussed above. The results of these studies and studies on other molecules are summarised in Table 1.

**Table 1:** Molecules tested in ovarian cancer models with MMP-14 and MMP-2

First author	Year	Model	Molecule(s) involved	Technique	Result
Kikkawa F [67]	2005	SKOV-3 cell line	Dipepeptidyl peptidase IV (DPPIV)	Immunoblotting	DPPIV decreases invasion, increases TIMP and E-cadherin
Barbolina M [38]	2006	DOV13 cell line	Epidermal Growth Factor Receptor (EGFR)	Q-PCR Zymography Three-dimensional collagen invasion assay	MMP-14 activation by collagen type I was investigated as measured by MMP-2 activation in zymography. This activation was induced only by EGR1 but not by EGR-2–4
Lin S-W [68]	2007	SKOV-3 cell line	Integrin -Linked Kinase (ILK)	Immunoblotting Zymography	ILK knockdown did not influence MMP-14 and MMP-2 expression and activity, but Smad-2 did via transforming growth factor-β1 (TGF-β1)
Cowden Dahl [69]	2007	OVCA 433 cell line	Epidermal Growth Factor Receptor (EGFR) Ets transcription factor PEA3	Q-PCR Zymography Matrigel invasion assay	Overexpression of the Ets transcription factor PEA3 induces EGFR-stimulated MMP-14 mRNA production
Devine K [70]	2008	DOV13 and OVCA 429 cell lines	Sphingosine-1-Phosphate (S1P) G-protein Gi	Zymography Western blot	Low S1P increases MMP-2 activity Gi-dependent, high S1P decreases MMP-14
Agarwal A [40]	2008	SKOV-3 and OVCAR-3 in nude mouse model	PAR1-based pepcudins	RT-PCR cell migration assays in vivo mouse model	Cell-penetrating pepducins targeting this cascade resulted in inhibition of PAR1-dependent angiogenesis, ascites and metastasis
Liao X [31]	2009	SKOV-3, OVCA 433 and OVCAR-3 cell lines	Hedgehog proteins Gli1	RT-PCR Matrigel invasion assay	Increased Gli1 expression is correlated with increased MMP-14 expression and proliferation, cell mobility and invasiveness, inhibitors of HH pathway proteins result in a reversed effect
Moss N [71]	2009	OVCA 433 cell line	Epidermal Growth Factor Receptor (EGFR)	Flow cytometry Western blot Three-dimensional collagen invasion assay	EGFR results in MMP-14 internalisation. Sustained surface MMP-14 causes enhanced migration and invasion
Jiang L [72]	2010	SKOV-3 and OVCA 420 cell lines	Factor Binding to the Inducer of short transcripts of human immunodeficiency virus-1 (FBI-1)	Western blot Matrigel invasion assay	FBI-1 activates MMP-14 by binding to its promotor and thereby enhancing its expression and the invasive properties of the cell
Poon S-L [73]	2011	OVCAR-3 and CaOV-3 cell lines	Gonadotropin-Releasing Hormone-II beta-catenin	Western blot Matrigel invasion assay	Gonadotropin-releasing hormone-II increases MMP-14 through the β-catenin signalling pathway and tincreases invasion
Nakayama I [74]	2013	OVCAR-3, JHOC-5,-7,-8, JHOS-2,-3,-4 and JHOM-1 cell lines	miRNA-10b (non coding RNA) and HOXD10 (mRNA encoding a transcriptional repressor)	RT-PCR Western blot Matrigel invasion assay	Up-regulation of miR-10b results in loss of HOXD10, induces MMP-14 expression and invasion in ovarian cancer cell lines
Yang Y [75]	2016	SKOV-3, A2780 and OMC685 cell line	Urothelial Carcinoma Associated 1 (UCA1) long noncoding RNA	Q-PCR Westen blot	UCA1 induces MMP-14 expression
Semprucci E [76]	2016	SKOV-3, HEY, CaOV-3 cell lines	Endothelin A receptor beta-arrestin/PDZ-RhoGEF	Confocal scanning laser microscopy	Endothelin A receptor regulates the function of invadopodia, resulting in MMP-related invasion through the β-arrestin/PDZ-RhoGEF pathway
Duan [77]	2018	SKOV-3, OVCAR-3 cell lines and mouse model	miR-122/P4HA1	Migration and invasion assays, Western blot as well as tumour spread in nude mouse model	miR-122 inhibited migration, invasion, EMT, and metastasis in peritoneal cavity of ovarian cancer cells by targeting P4HA1

#### Synopsis of findings on MMP-14 in ovarian cancer in basic research

In ovarian cancer, MMP-14 seems to play an important role in invasion and metastasis as well as in proliferation and angiogenesis, though the latter two processes have been less well studied. In most studies with a controlled design, the presence or expression of MMP-14 led to more invasion and metastasis as well as proliferation and angiogenesis. The growth and spread of ovarian cancer can be orchestrated by MMP-14 in conjunction with other molecules including MMP-2, TIMP-2 and the integrins. However, none of these players including MMP-14 seems to be essential as invasion can also occur without the influence of MMP-14 but is then usually less extensive [42, 44]. Differences between the studies discussed above may mostly be attributable to the models and materials used in the studies.

### MMP-14 and ovarian cancer in clinicopathological research

Although most basic research studies have a controlled design, translation of the findings in cell lines to patients is not straightforward. MMP-14 has been studied in patient material with various techniques. The first studies performed were with the technique of in-situ hybridization (ISH). MMP-14 has also been determined in serum. One study investigated MMP-14 polymorphisms [50]. However, most studies have been performed with IHC and compared benign tumours, borderline ovarian tumours and ovarian cancer.

#### In-situ hybridization

Initially, Afzal et al. investigated MMP˗14 mRNA in-situ hybridization (ISH) in different type ovarian tumours [51]. Few benign tumours, but six borderline tumours showed MMP-14 mRNA expression. Of the malignant tumours, only one was negative for tumour cells and stroma, and 18/19 showed MMP-14 mRNA expression in the stroma, of which 8/19 also showed expression in the epithelium.

The first study on MMP-14 mRNA expression and survival was by Davidson et al.[52] They investigated 45 patients, of which 21 were long-term survivors (mean disease-free survival of 109 months (DFS) and mean overall survival (OS) of 125 months), and 24 were short-term survivors (mean DFS three months and mean OS of 21 months). MMP-14 mRNA expression in tumour cells of the metastasis correlated with poor survival, while expression in the stromal cells correlated with longer survival. These findings have contributed to the hypothesis that MMP-14 on the tumour cells indicates more aggressive tumour characteristics and that MMP-14 in the stroma is part of the anti-tumour response by the host. In a follow-up study also considering angiogenic factors, no correlation between MMP-14 and angiogenic factors was found [53]. Part of the cohort was also studied for correlation between the MMPs and integrins, as well as correlation between Ets transcription factors and MMP-14 [54]. This study suggested that MMP-synthesis occurs after integrin activation. However, these findings in tumour tissue are not in line with the basic research on the DOV13 cell line [35, 36].

Davidson et al. also studied a different cohort, where the results for reverse transcriptase polymerase chain reaction (RT-PCR) and ISH correlated well [55]. No differences in expression were found between peritoneal and pleural effusions suggesting that the cells in peritoneal and pleural effusions are similar.

#### Serum MMP-14

Serum MMP-14 was determined in a small series of fluids (cystic, ascites and pleural effusions) from 14 patients, 10 of them being malignant and demonstrating a threefold increase compared to benign fluids. In a series of 92 serum samples, 26 from patients with ovarian cancer, MMP-14 was found to be significantly increased in the ovarian cancer patients compared to those with benign masses or healthy females [44]. This is an interesting finding, given the increased ectodomain shedding by increased expression of MMP-14 of MUC16/CA 125 [49].

#### Immunohistochemistry

For an overview of the relevant clinicopathological studies using IHC and their main findings, see Table 2. Details about the studies can be found in this table.

**Table 2:** Results of immunohistochemistry on MMP-14 in ovarian cancer

Author	Tumour samples	Selection of samples	Years	Country	Scoring system	Number of patients or samples	Result MMP-14	Percentage positive	Conclusion
Sakata et al. [56]	Primary tumours	Selection from archive UH^a^	n.m	Japan	0–5% = 0, 5–50% = 1, > 50% = 2	114	0 benign, 5 borderline, 59 malignant	76%	More MMP-14 expression in tumours with lymphnode metastasis
Torng et al. [64]	Primary tumours, serous and endometroid	Selection from archive UH^a^	1993–2000	Taiwan			positive, numbers n.m.^b^		Expression MMP-14 and MMP-2 correlates
Kamat et al. [57]	Primary tumours	Selection from archive UH^a^	1990–2000	USA	Overall Score	90	90 epithelial	100, moderate 56%, strong 44%	HR^c^ epithelial MMP-14 2,52 (1.30–4.88)
							87 stromal	97 (62% low, 38% high)	HR^c^ stromal MMP-14 1,30 (1.03–3.39)
									Both MMP-14 epithelial and stromal expression correlates with survival
Lin et al. [59]	Primary tumours	Selection from archive UH^a^	n.m	USA		77		44% epithelial expression	Strong epithelial MMP-14 expression > 3 risk of death p 0.003
								40% stromal expression	
Barbolina et al. [38]	Primary tumours	Selection from archive UH^a^	n.m	USA		149		78%, 52% high, 94% in clear cell carcinomas	No relation with stage
Adley et al. [26]^d^	Primary tumours	Selection from archive UH^a^	1999–2003	USA		143	77	54	32/70 serous, 23/44 endometroid, 5/9 mucinous, 17/18 clear cell
Paulsen et al. [63]	Primary serous borderline tumours and implants	Selection from Cancer Registry (total 632)	1985–1995	Norway		Serous borderline tumours	7 moderate, 33 strong/44, 4 weak, 8 moderate, 42 strong/55		No difference between group with and without implants, all 7 recurrences positive
Brun et al. [60]	Primary serous and mucinous tumours	Selection from archive UH^a^	2001–2006	France	HSCORE	Serous	115	benign/borderline 99/95, malignant 134	
						Mucinous	44		
Kato and Motoyama [65]	Primary clear cell and serous carcinomas	Selection from archive UH^a^	1997–2007	Japan		30 clear cell carcinomas	22	73	No relation with stage or survival
						30 serous carcinomas	1	3	
Wang et al. [78]	Primary tumours and metastasis	Selection from archive UH^a^	2001–2006	China				positive, numbers n.m.^c^	Colocalization with uPA, numbers not given
Moss et al. [32]	Primary tumours and metastasis	Selection from archive UH^a^	n.m.^b^	USA	0–3	15 serous primary	5 weak, 9 moderate, 1 strong	67	
						15 serous metastasis	4 weak, 9 moderate, 2 strong	73	Only 2 metastasis had less expression than the primary tumour
						2 endometroid	1 weak	50	
						2 endometroid metastasis	2 moderate	100	
Brun et al. [60]	Primary tumours	Consecutive series UH^a^ FIGO III-IV	2001–2006	France	HSCORE	43 primary surgery	132 epithelial/12 stromal		No independent prognosticator
						26 interval surgery	97 epithelial/8 stromal		
Bruney et al. [49]	Cores of ovarian adenocarcinomas	N.m.^b^	n.m	USA		50	positive, numbers n.m.^b^		Inverse relationship with MUC16/CA 125
Nakayama et al. [74]	Primary tumours	Selection from archive UH^a^	2005–2011	Japan		68	12	18	
Trudel et al. [79]	Primary tumours	Selection from archive UH^a^	1993–2006	Canada	Visual score HSCORE	160/170	105	66	No independent prognosticator
Bruney et al. [48]^e^	Cores of ovarian adenocarcinomas	N.m.^b^	n.m	USA		46	positive, numbers n.m.^b^		
Vos et al. [61]	Primary tumours	Consecutive series archives teaching hospital	1997–2003	the Netherlands	Overall Score	94	53 positive epithelial	56	No independent prognosticator
							49 positive stromal	52	
Vos et al. [62]	Primary tumours	Consecutive series archives clinical network	2007–2008	the Netherlands	Overall Score	97	52 positive epithelial	54	
							5 positive stromal	5	
Takahashi et al. [80]	Primary tumours	Selection from archive UH^a^	2000–2014	Japan	Visual and computer-supported evaluation	107 carcinoma's, 54 borderline, 45 benign	positive, numbers n.m.^b^		In-situ proximity assay for EphA2 and MMP-14 positive
Vos et al. [58]	Primary tumours and haematogenic and lymphogenic metastasis	Consecutive series archives teaching hospital	2000–2015	the Netherlands	Overall Score	44	37 primary tumours positive/7 missing	37/44	Most metastasis also positive
							34 positive stromal/3 negative stromal/7 missing		

^a^*UH* University Hospital

^b^*n.m.* Not mentioned

^c^*HR* Hazard Ratio

^d^overlap with study by Barbolina not clear

^e^overlap with earlier Bruney study not clear

In most studies, malignant and borderline tumours, showed higher MMP-14 expression than benign tumours [56]. Various studies showed a correlation with poor prognostic factors such as high stage and high grade. Strong epithelial MMP-14 expression and high stromal MMP-14 were significant factors in multivariate analysis in one study [57]. In our study on lymphogenic and hepatogenic metastasis, all tumours were MMP-14 positive [58].

Sometimes a correlation with survival is found [59]. However, in a study by Brun et al., the expression of epithelial MMP-14 appeared to predict survival but this effect disappeared after Bonferroni correction [60]. We did also not find that MMP-14 was an independent prognostic factor [58, 61, 62].

Paulsen et al. investigated 99 serous borderline ovarian tumours, of which 44 had non-invasive implants. The results were not significantly different between the non-invasive implant group and the group without implants. All samples from the seven patients who relapsed, were strongly positive for MMP-14 [63]. Moss et al. studied 17 patients in which the peritoneal metastasis showed either similar or increased MMP-14 expression compared to the primary tumour [32].

In the studies, where both MMP-14 and MMP-2 were studied, their expression mostly correlated [61, 64].

Barbolina et al. demonstrated that the highest expression of MMP-14 was in clear-cell carcinomas (94%) [38]. Clear-cell carcinomas showed high expression with 17/18 samples with positive expression compared to the other histiotypes, in which expression was 45–55% in another study by the same group [26]. However, the overlap between these studies was not clear. A study from Japan confirmed high MMP-14 expression in 22/30 clear cell carcinomas, while only 1/30 serous carcinomas was positive without correlation with stage or survival [65]. In a French study, serous tumours, especially the malignant ones, showed higher MMP-14 expression than the mucinous tumours [60].

#### Synopsis of findings on MMP-14 IHC in ovarian cancer

The wide range of MMP-14 expression measured in these studies may be explained by several factors. One is the use of a variety of antibodies and the fact that most of them were polyclonal. The disadvantage of using polyclonal antibodies is that they bind not only to intact or active MMP-14 but also to its degraded products. Therefore, more background staining is to be expected.

A second possible explanation for the wide range of results is the use of several different scoring systems for MMP-14 expression, most of which were semi-quantitative, which may result in a low discriminative value. Unfortunately, no large validation studies on these scoring systems have yet been conducted. The use of the HSCORE seems a promising tool due to the use of an objective count and possible wider range of scores [64].

A third possible factor is non-uniformity and lack of clarity in the selection of patient cohorts. As some of the studies do not report the selection criteria used, this aspect is impossible to investigate.

Finally, limited data are available on overall and disease-free survival outcomes. In the review on MMP-14 expression on cancer in general [15], MMP-14 was an independent prognosticator. For ovarian cancer however, the data on survival are limited. In the few studies that are available on survival, MMP-14 is related to prognostic factors and (disease-free) survival, but its independent value turns out to be limited after correction for known prognostic factors or statistical correction for the number of tests.

## Conclusion

From this systematic review of current knowledge regarding the role of MMP-14 in ovarian cancer, MMP-14 comes to the forefront as an important molecule in the pathophysiology of ovarian cancer. Basic research shows that it plays a role in proliferation, invasion, angiogenesis and metastasis. However, MMP-14 does not seem to be an essential player because in some studies invasion is possible without MMP-14, though in the presence of MMP-14 invasion and metastasis is more extensive. Although the development of advanced tumours is mediated through several pathways, MMP-14 expression occurs in most advanced-stage ovarian cancers and not in all early-stage tumours. The limited available research on survival indicates that MMP-14 is not an independent predictor for prognosis. This may be due to several limitations in the studies, but also to the fact that in ovarian cancer other prognostic factors may overrule the role of MMP-14.

Therefore, we recommend larger validation studies of the IHC scoring systems for MMP-14 in order to determine the predictive role of MMP-14 more precisely. However, the heterogeneity of expression within the tumour may still be a limiting factor.

Ideally, monoclonal antibodies are used for these IHC studies and also tested for their therapeutic effect in MMP inhibition. In the past, generalized MMP-14 inhibition has not shown to be effective. By identifying the precise mechanisms whereby MMP-14 stimulates tumour growth, anti-MMP agents that have a targeted action are developed and can be tested in a minimal residual disease setting after debulking.

Given the widespread presence of MMP-14 in advanced-stage disease, these new targeted agents may be used in spite of the limited prognostic value of MMP-14. If a given tumour is MMP-14 positive irrespective of its histological type, targeted treatment can be administered after debulking with chemotherapy and surgery if necessary and hopefully lead to prolonged responses to treatment. A promising new development is a MMP-14 specific nanoprobe that facilitates in vivo detection of MMP-14 tumour cells. This nanoprobe can be used during debulking surgery and therefore be an attractive therapeutic tool for success of debulking surgery [66].

## Supplementary Information

**Additional file 1: Appendix 1**. Search strategy for MMP-14 and ovarian cancer.

## Data Availability

Not applicable.
