# Supplementary material for: The role of MMP-14 in ovarian cancer: a systematic review
Source: J Ovarian Res. 2021 Aug 3;14:101. doi: 10.1186/s13048-021-00852-7 (PMC8336022; doi:10.1186/s13048-021-00852-7)
Supplement: Supplementary file 1 — Additional file 1: Appendix 1. Search strategy for MMP-14 and ovarian cancer. [file 13048_2021_852_MOESM1_ESM.pdf]

Appendix 1. Search strategy for MMP-14 and ovarian cancer.

#12	Add	Search **(#11) AND #9**	92
#11	Add	Search **(Ovarian Neoplasms [Mesh]) OR (Ovarian Neoplasm [tiab] OR Ovary Neoplasms [tiab] OR Ovary Neoplasm [tiab] OR Ovary Cancer [tiab] OR Ovary Cancers [tiab] OR Ovarian Cancer [tiab] OR Ovarian Cancers [tiab] OR Cancer of Ovary [tiab] OR Cancer of the Ovary [tiab] OR tumors of the ovaries [tiab] OR Tumor of the ovary [tiab] OR Tumors of the Ovary [tiab] OR Tumors of the ovary [tiab] OR ovarian neoplasm [tiab] OR ovary neoplasms [tiab] OR ovary neoplasm [tiab] OR ovary cancers [tiab] OR ovarian cancer [tiab] OR ovarian cancers [tiab] OR cancer of ovary [tiab] OR cancer of the ovary [tiab] OR tumor of the ovary [tiab] OR tumors of the ovary [tiab] OR tumours of the ovary [tiab] OR tumour of the ovary [tiab])**	116350
#9	Add	Search **((MMP-14 Metalloproteinase [TIAB] OR MMP 14 Metalloproteinase [TIAB] OR MT1-MMP [TIAB] OR MMP14 Metalloproteinase [TIAB] OR Metalloproteinase, MMP14 [TIAB])) OR (Matrix Metalloproteinase 14"[Mesh] OR Metalloproteinase 14, Matrix [TIAB] OR 'MMP-14 Metalloproteinase' [TIAB] OR MMP 14 Metalloproteinase [TIAB] OR Metalloproteinase, MMP-14 [TIAB] OR MMP14 Metalloproteinase [TIAB] OR MT-MMP-1 [TIAB] OR MT1-Matrix Metalloproteinase [TIAB] OR MMP-14 [tiab]))) AND (Search ("Matrix Metalloproteinase 14"[Mesh] OR Metalloproteinase 14, Matrix [TIAB] OR 'MMP-14 Metalloproteinase' [TIAB] OR MMP 14 Metalloproteinase [TIAB] OR Metalloproteinase, MMP-14 [TIAB] OR MMP14 Metalloproteinase [TIAB] OR MT-MMP-1 [TIAB] OR MT1-Matrix Metalloproteinase [TIAB] OR MMP-14 [tiab])**	5092
